# Skeletal Stability after Mandibular Setback via Sagittal Split Ramus Osteotomy Verse Intraoral Vertical Ramus Osteotomy: A Systematic Review

**DOI:** 10.3390/jcm10214950

**Published:** 2021-10-26

**Authors:** Chun-Ming Chen, Dae-Seok Hwang, Szu-Yu Hsiao, Han-Sheng Chen, Kun-Jung Hsu

**Affiliations:** 1 School of Dentistry, College of Dental Medicine, Kaohsiung Medical University, Kaohsiung 80708, Taiwan; komschen@yahoo.com.tw (C.-M.C.); syhsiao2004@yahoo.com.tw (S.-Y.H.); 2 Department of Oral and Maxillofacial Surgery, Kaohsiung Medical University, Kaohsiung 80708, Taiwan; 3 Department of Oral and Maxillofacial Surgery, Pusan National University Dental Hospital, Pusan 50612, Korea; dshwang@pusan.ac.kr; 4 Department of Dentistry for Child and Special Needs, Kaohsiung Medical University Hospital, Kaohsiung 80708, Taiwan; 5 Dental Department, Kaohsiung Municipal Siaogang Hospital, Kaohsiung 80812, Taiwan; 6 Department of Dentistry, Kaohsiung Medical University Hospital, Kaohsiung 80756, Taiwan

**Keywords:** skeletal stability, mandibular setback, sagittal split ramus osteotomy, intraoral vertical ramus osteotomy

## Abstract

Purpose: The purpose of present study was to review the literature regarding the postoperative skeletal stability in the treatment of mandibular prognathism after isolated sagittal split ramus osteotomy (SSRO) or intraoral vertical ramus osteotomy (IVRO). Materials and Methods: The articles were selected from 1980 to 2020 in the English published databases (PubMed, Web of Science and Cochrane Library). The articles meeting the searching strategy were evaluated based on the eligibility criteria, especially at least 30 patients. Results: Based on the eligibility criteria, 9 articles (5 in SSRO and 4 in IVRO) were examined. The amounts of mandibular setback (B point, Pog, and Me) were ranged from 5.53–9.07 mm in SSRO and 6.7–12.4 mm in IVRO, respectively. In 1-year follow-up, SSRO showed the relapse (anterior displacement: 0.2 to 2.26 mm) By contrast, IVRO revealed the posterior drift (posterior displacement: 0.1 to 1.2 mm). In 2-year follow-up, both of SSRO and IVRO presented the relapse with a range from 0.9 to 1.63 mm and 1 to 1.3 mm respectively. Conclusion: In 1-year follow-up, SSRO presented the relapse (anterior displacement) and IVRO posterior drift (posterior displacement). In 2-year follow-up, both of SSRO and IVRO showed the similar relapse distances.

## 1. Introduction

Currently, a multimethod approach of orthognathic surgery [1,2,3,4,5,6,7,8] is used to correct mandibular prognathism. The most commonly performed surgeries are sagittal split ramus osteotomy (SSRO) and intraoral vertical ramus osteotomy (IVRO). As indicated by Wolford [9], the benefit of SSRO is that it can accelerate and strengthen the bone healing process by creating larger overlapping bone segments and incorporating a rigid fixation method. After surgery, patients are able to open their mouth; the airway is more likely to remain unimpeded, thus improving their speaking condition and oral hygiene. Furthermore, the mandible can be moved immediately after the surgery, which enables patients to maintain the required nutrition in the early postoperative period and consume normal food sooner. Accordingly, SSRO increases patients’ comfort after surgery and facilitates their postoperative orthodontic treatment. However, Wolford [9] also mentioned two main drawbacks of SSRO. First, the chance of injury to the mandibular alveolar nerves is higher; thus, patients have a higher risk of experiencing neurosensory disturbance in the lower lip following surgery. Second, if the condyle is inaccurately positioned in the articular fossa during the operation, immediate occlusion shifts will occur postoperatively. In mild cases, the sequelae of malocclusion can be improved through postoperative orthodontic treatment; however, in severe cases, patients must undergo condylar repositioning. 

In contrast, according to the report of Ghali [10], IVRO has two main benefits. First, the incidence of nerve damage is much lower than that in SSRO. Second, rigid or semirigid fixation is not required after IVRO. Therefore, the condyle enters a new equilibrium position, and the range of motion of the mandible can recover more naturally. The contributing factors to skeletal relapse after mandibular setback surgery include the surgical method (SSRO or IVRO), the area of the pterygomasseteric sling’s detachment, the fixation method (proximal and distal segment with or without rigid fixation), and the amount of mandibular setback, etc. Therefore, the aim of our systematic review was to investigate the factors affecting postoperative skeletal stability between SSRO and IVRO in the treatment of mandibular prognathism.

## 2. Materials and Methods

### 2.1. Search Strategy

A systematic search of English-language databases, including PubMed, Web of Science, and Cochrane Library, was conducted. Studies from 1980 to 2020 with medical subject headings and their synonyms as keywords, such as “sagittal split ramus osteotomy”, “intraoral vertical ramus osteotomy”, “mandibular prognathism”, “mandibular setback”, and “stability”, were collected. Moreover, relevant articles from the references of the selected articles were also evaluated. 

### 2.2. Study Selection and Eligibility

The eligibility criteria for the literature review were as follows: (1) being a randomized controlled trial, case series, or observational study; (2) having at least 30 patients with mandibular prognathism; (3) involving only mandibular SSRO or IVRO; (4) having cephalometric analysis with B point, Pog, and Me as landmarks; and (5) having a 1-year postoperative follow-up. Based on the eligibility criteria, two authors retrieved and selected articles for full-text reading; consequently, they evaluated the titles and abstracts of the studies. The following articles were excluded: case reports, reviews, and studies involving patients with prior facial trauma or a history of facial surgery.

### 2.3. Data Extraction and Analysis of Surgical Stability

Information regarding methodological quality, patient demographics, and postoperative stability data was independently evaluated by two authors. The referential landmarks (B point, Pog, and Me) were used to present the postoperative stability and to analyze the changes in horizontal distances. This article was written according to the PRISMA (Preferred Reporting Items for Systematic Reviews and Meta-Analyzes) statement [11].

## 3. Results

### 3.1. Data Consolidation Analysis

**Figure 1:**
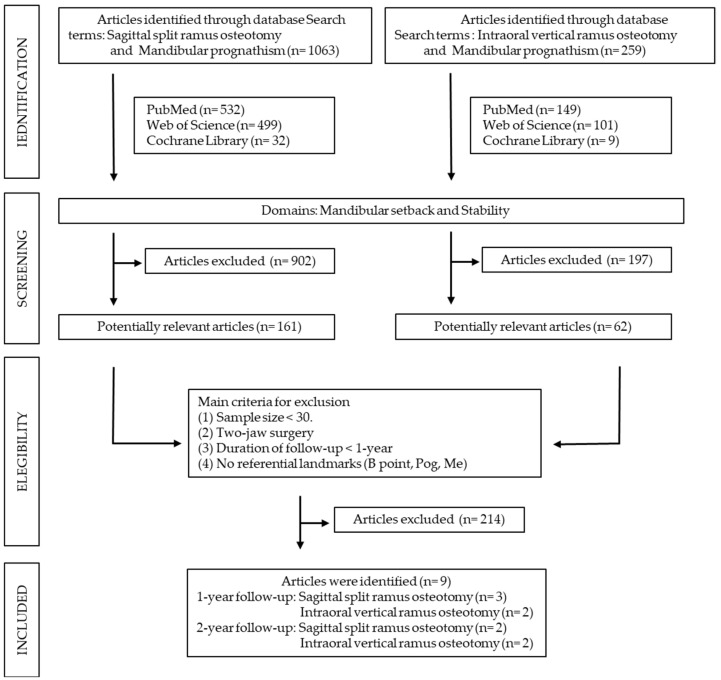
Process flow of article selection in sagittal split ramus osteotomy and intraoral vertical ramus osteotomy.

A total of 1063 articles were retrieved using the search terms “sagittal split ramus osteotomy” and “mandibular prognathism” in PubMed (*n* = 532), Web of Science (*n* = 499), and Cochrane Library (*n* = 32) databases (Figure 1). Of these, 161 articles were retained by further narrowing to the domains of “mandibular setback” and “stability” (PubMed, *n* = 73; Web of Science, *n* = 80; Cochrane Library, *n* = 8). For IVRO, 259 articles were retrieved using the search terms “intraoral vertical ramus osteotomy” and “mandibular prognathism” in PubMed (*n* = 149), Web of Science (*n* = 101), and Cochrane Library (*n* = 9) databases (Figure 1). Of these, 62 articles were retained by further narrowing to the domains of “mandibular setback” and “stability” (PubMed, *n* = 23; Web of Science, *n* = 36; Cochrane Library, *n* = 3). A total of 902 and 197 articles were excluded from the SSRO pool and IVRO pool, respectively. As a result, 223 articles (161 in SSRO and 62 in IVRO) were shortlisted for further screening and selection.

### 3.2. Study Selection and Eligibility

**Figure 2:**
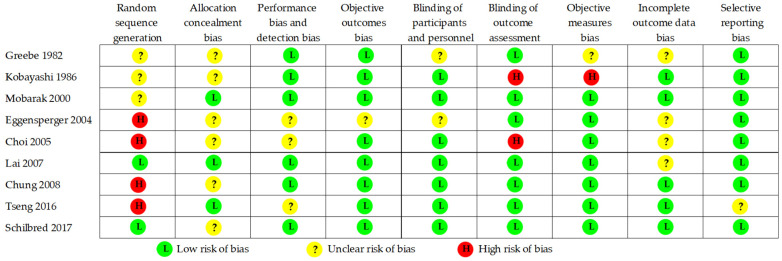
Risk of bias summary.

**Figure 3:**
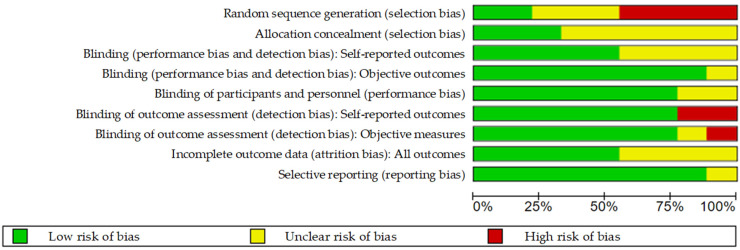
Risk of bias graph.

Two authors independently reviewed and retrieved the titles and abstracts of the 223 articles. To qualify the inter-rater reliability, a kappa coefficient test was performed. The kappa value was 0.870 (*p* < 0.001), revealing high consistency between the two authors. The following studies were excluded: (1) in vitro and animal studies; (2) studies with duplicated or incomplete data (no referential landmarks: B point, Pog, Me); (3) having very small sample size (*n* < 30) and two-jaw surgery; and (4) not having a post-operative follow-up of at least 1 year, which is necessary for skeletal remodeling and stability. Therefore, a total of 156 articles in SSRO and 58 articles in IVRO were excluded. Finally, the remaining nine articles [12,13,14,15,16,17,18,19,20] (five in SSRO and four in IVRO) were selected and investigated (Table 1). A total of 445 patients with mandibular prognathism were treated using SSRO (300 patients) or IVRO (145 patients). As shown in Figure 2, nine articles were evaluated for the risk of bias. Figure 3 presents a summary of the risk of bias. Sequence generation bias was 44.4% (4/9) in the high risk, while the selective reporting bias was 88.9% (8/9) in the low risk.

### 3.3. Data Extraction and Analysis of Surgical Stability

All SSRO and IVRO patients had received preoperative and postoperative orthodontic treatments. For intersegment fixation, three studies used miniscrews and one study used wire to carry out interosseous fixation between the proximal and distal segments in SSRO. However, most patients with SSRO still required elastic maxillomandibular fixation from 1 to 6 weeks. On the contrary, no fixation between the proximal and distal segments was required in IVRO. However, a 6-week maxillomandibular fixation by wire was necessary for IVRO. In the 1-year follow-up, SSRO and IVRO had three and two articles, respectively. The amount of setback (B point, Pog, and Me) in SSRO and IVRO ranged from 5.53 to 9.07 mm and 6.7 to 13.3 mm, respectively. In the 2-year follow-up, both SSRO and IVRO had two articles, and the amount of setback (B point and Pog) ranged from 6.28 to 8.2 mm and 8.3 to 12.4 mm, respectively, in SSRO and IVRO. In SSRO, all articles presented relapse (anterior displacement) with a range of 0.2–2.26 mm in the 1-year follow-up. However, the articles on IVRO (1-year follow-up) revealed posterior drift (posterior displacement) with a range of 0.1–1.2 mm. In the 2-year follow-up, the articles on SSRO still showed relapse with a range of 0.9–1.63 mm. Similarly, relapse occurred in IVRO with a range of 1–1.3 mm.

## 4. Discussion

### 4.1. Risk of Bias Assessment

From our observation, four out of nine articles (44.4%) revealed no data collection period. We considered a high risk of bias for sequence generation, and most of the articles (66.7%) showed unclear information for keeping the surgeon(s) and participants unaware of the sequence. Analyzing judgments for performance bias, we found that the blinding of participants and personnel was 77.8% in the low risk of bias. All articles were deliberately, completely, and accurately reported. The selective reporting bias was 88.9% in the low risk of bias. Therefore, all eligible articles have a certain reference value for the assessment of skeletal stability after mandibular setback via SSRO versus IVRO. Postoperative stability following SSRO and IVRO was discussed through the following aspects based on reports in the literature.

### 4.2. Detachment of Pterygomandibular Sling

From an anatomical perspective, two main differences were found between IVRO and SSRO in the treatment of patients with mandibular prognathism. First, the degree of detachment in the pterygomandibular sling (masseteric and medial pterygoid muscles) was greater in IVRO than in SSRO. Therefore, the stretching of the pterygomandibular sling is different when the mandible (distal segment) is set back. SSRO tends to stretch the medial pterygoid muscle backward; concurrently, the masseteric muscle is not detached as long as the proximal segment moves behind the masseteric muscle, and thus the sling is stretched, thereby increasing the risk of relapse. In IVRO [4,5], the masseteric muscle is completely detached from the lateral surface of the ramus, and most of the medial pterygoid muscle is detached from the medial surface of the ramus. To preserve a small portion of the medial pterygoid muscle attached to the proximal segment, it is used to seat the condyle in the glenoid fossa. Therefore, the risk of pterygomandibular sling stretching is lower in IVRO than in SSRO when the distal segment is set back, thus reducing the chance of relapse. Second, IVRO cuts through the posterior ramus, and SSRO splits the entire ramus in half. Therefore, wound healing differs between IVRO and SSRO. The patterns of bone healing and remodeling are cortex-to-cortex in IVRO and marrow-to-marrow in SSRO. Therefore, SSRO presented relapse (anterior displacement: 0.2 to 2.26 mm) [13,14,16] and IVRO presented posterior drift (posterior displacement: 0.1 to 1.2 mm) [15,17] in the 1-year follow-up. After the 2-year follow-up, bone healing and remodeling tended towards stability. Both SSRO and IVRO showed similar relapse distances of 0.9–1.63 mm [12,18] and 1–1.3 mm [19,20], respectively.

### 4.3. Condylar Sag

Condylar sag often occurs after IVRO [4,5] due to the detachment of the masseteric and medial pterygoid muscles such that the condyle is affected by gravity and the pull of the lateral pterygoid muscle, resulting in anteroinferior displacement. Moreover, IVRO procedures do not implement proximal and distal segment fixation, leading to the occurrence of postoperative condylar sag. In contrast, SSRO retains the attachment of the medial pterygoid muscle and the stylomandibular ligament to the posterior border of the proximal segment and uses rigid fixation between the proximal and distal segments. Therefore, the condyle is easily positioned posterosuperiorly, and condylar sag is seldomly seen after SSRO. 

### 4.4. Proximal and Distal Segment Fixation

There are different designs for proximal and distal segment fixation between SSRO and IVRO. SSRO usually uses rigid (miniscrew or miniplate) [14,15,16,18] or semirigid fixation (wire) [13] for interosseous fixation between the proximal and distal segments. Politi et al. [21] investigated postoperative skeletal stability between rigid (miniplates and screws) and semirigid fixation (wire osteosynthesis and maxillomandibular fixation for 6 weeks) for the correction of skeletal Class III malocclusion. No significant differences in postoperative skeletal and dental stability were observed between the rigid and semirigid groups. Rigid fixations involve various materials and techniques, such as monocortical osteosynthesis, bicortical osteosynthesis, miniplate–miniscrew, resorbable miniscrew, and miniplate. Hsu et al. [22] evaluated the postoperative stability between bicortical and monocortical osteosynthesis in the treatment of mandibular prognathism. They reported that the sagittal relapse rate was 20% in the bicortical group and 25% in the monocortical group. However, both groups had no statistically significant differences in postoperative stability. Chung et al. [18] examined the postoperative stability with monocortical plate fixation or bicortical screw fixation after SSRO for mandibular prognathism. They [18] reported no statistically significant differences between monocortical plate and bicortical screw fixation. Ueki et al. [23] compared the skeletal stability between monocortical plate, bicortical plate, and hybrid fixation techniques using absorbable plates and screws; however, there were no significant differences in the postoperative skeletal stability among the three groups. 

In contrast, IVRO rarely uses rigid or semirigid fixation for interosseous fixation between the proximal and distal segments because of two main reasons. First, IVRO does not require rigid or semirigid fixation to achieve postoperative stability. Athanasiou et al. [24] conducted extraoral vertical ramus osteotomy in 52 patients and performed proximal and distal segment fixation using wires in 26 patients and no wires in the other half. No significant difference was observed in the postoperative skeletal stability with or without the use of a wire. Second, the implementation of rigid or semirigid fixation has some disadvantages in IVRO, including technical difficulties, prolonged operation time, and the need for a small external incision on the cheek. In the extraoral or IVRO, the proximal and distal segments need not be fixed by wire because the postoperative restoration of muscle tone will maintain the position of the condyle within the glenoid fossa. 

### 4.5. Maxillomandibular Fixation

SSRO uses rigid and elastic fixation for maxillomandibular fixation (MMF) (1 to 6 weeks). Harada et al. [25,26] evaluated postoperative stability in prognathic patients with symmetric and asymmetric mandibles under SSRO without postoperative MMF. They reported that postoperative MMF may be avoided in both symmetric and asymmetric mandibles. Yamada et al. [27,28] investigated the postoperative course after SSRO in mandibular asymmetries with or without MMF. The report revealed that postoperative skeletal stability was satisfactory in both groups, and there was no correlation between the surgical results and use of postoperative MMF. Considering the risks of airway distress, Yamada et al. [27,28] recommended that MMF is not necessary after rigid fixation SSRO, even for mandibular asymmetry. Owing to the lack of fixation between the proximal and distal segments, a 6-week MMF was applied for mandible immobilization after IVRO. Al-Delayme et al. [29] compared the postoperative skeletal stability after IVRO without fixation and SSRO with rigid fixation (miniplate), which took 6 to 8 weeks of MMF for both IVRO and SSRO. They [29] found that the percentage of relapse after IVRO was similar to that after SSRO. We noted that Kobayashi et al. performed SSRO with 6 weeks of MMF and attained good skeletal stability. Even with semirigid (wire) fixation between the proximal and distal segments, Pog and Me showed insignificant relapse by 0.2 and 0.4 mm, respectively. The postoperative skeletal stability of Kobayashi et al. was better than that of other authors [14,15,16,18]. Investigating the duration of MMF in SSRO, Chung et al. [18] used an elastic (4 to 5 days) and revealed a greater percentage of relapse in Pog and Me (24% and 28.9%, respectively) than in others [12,14,15,16].

### 4.6. Amount of Setback

Takahara et al. [30] investigated postoperative skeletal relapse in terms of the effects brought about by the magnitude of mandibular setback in SSRO. They reported that increased relapse was associated with greater mandibular setback and increased proximal segment clockwise rotation. Yang and Hwang [31] analyzed possible contributing factors to intraoperative clockwise rotation of the proximal segment by SSRO. They also revealed that patients with large clockwise rotation showed a significantly greater tendency towards skeletal relapse than patients with small clockwise rotation. In contrast to previous reports, Chen et al. [32] showed that there was a significant correlation between smaller amounts (≦8 mm) of mandibular setback and no correlation between larger amounts (>8 mm). In IVRO, Choi et al. [33] reported that the amount of setback can be a key factor in predicting postoperative mandibular relapse. They also found that the amount of setback decreased and the mandibular posterior drift increased after IVRO. Tseng et al. [34] reported that a significant relapse was correlated with the clockwise rotation of the distal segment. Investigating the postoperative stability of conventional SSRO and surgery-first SSRO, Mah et al. [35] reported that a greater horizontal and vertical relapse may occur because of counterclockwise rotation of the mandible in surgery-first SSRO. Ko et al. [36] also found that the amount of surgical setback, overbite (positive values), overjet, and depth of the curve of Spee showed statistically significant correlations with the amount of relapse in surgery-first SSRO.

Nevertheless, the results of the present study show that SSRO and IVRO have good postoperative skeletal stability. However, this study has some limitations. First, there were only nine articles included (SSRO: five articles; IVRO: four articles), which were not evident enough to provide clinical consideration. Another limitation was that the selected articles conducted two-dimensional cephalometric analysis. Further research should perform a 3D cephalometric analysis of postoperative skeletal stability.

## 5. Conclusions

Through the literature review concerning the stability of SSRO and IVRO for mandibular setback, nine articles (five in SSRO and four in IVRO) were selected and retrieved based on the eligibility criteria. Due to differences in the surgical manipulations and proximal–distal segment fixation methods, we concluded the following:

(1) The amount of mandibular setback (B point, Pog, and Me) ranged from 5.53 to 9.07 mm in SSRO, and skeletal relapse revealed anterior displacement (0.2 to 2.26 mm) in the 1-year follow-up.

(2) The amount of mandibular setback (B point, Pog, and Me) ranged from 6.7 to 12.4 mm in IVRO, and posterior drift (0.1–1.2 mm) was found in the 1-year follow-up.

(3) In the 2-year follow-up, both SSRO and IVRO presented good postoperative skeletal stability. The relapse distances of SSRO and IVRO were 0.9–1.63 mm and 1–1.3 mm, respectively.

## Figures and Tables

**Table 1:** Demographic and study characteristics in the included studies.

Author	Techniques	Samples	Age	Sex	Pre- & Post-	Proxmial-Distal	Maxillo-	Follow-Up	Setback	Postoperative Displacement			
Year			Mean (Years)	F (Female)	Surgical	Segment	Mandibular	Mean	(mm)	≥1 Year		≥2 Year	
Country of Origin			Range (Years)	M (Male)	Orthodontic	Fixation	Fixation (Weeks)	(Months)		(mm, %)		(mm, %)	
Greebe and Tuinzing [12]	IVRO	*n* = 35	NA	NA	Presurgical (+)	No fixation	6 (wire)	12	Pog 7.5	+1.2, 16%	NA		
1982					Postsurgical (+)				B point 6.7	+0.8, 11.9%	NA		
Netherlands													
Kobayashi et al. [13]	SSRO	*n* = 44	19.5F	34F	Presurgical (+)	wire	6 (wire)	12	B point 8.4	−0.6, 7.1%	*		
1986			21.7M	10M	Postsurgical (+)				Pog 8.4	−0.2, 2.4%	―		
Japan			16–27						Me 8.5	−0.4, 4.7%	―		
Mobarak et al. [14]	SSRO	*n* = 80	24.8	34F	Presurgical (+)	miniscrew	2–4 (elastic)	36	B point 6.93			−1.27, 18.3%	*
2000			17.6–51	46M	Postsurgical (+)				Pog 6.28			−1.63, 26%	*
Noway													
Eggensperger et al. [15]	SSRO	*n* = 30	23.5	NA	Presurgical (+)	miniscrew	≤1 (wire)	14	B point 5.97	−0.77, 12.9%	―		
2004					Postsurgical (+)				Pog 5.53	−0.5, 9%	―		
Switzerland									Me 6.03	−0.9, 14.9%	*		
Choi et al. [16]	SSRO	*n* = 86	24	57F	Presurgical (+)	miniplate (*n* = 15)	6 (elastic)	24	Pog 8.2			−1.1, 13.4%	NA
2005			16–43	29M	Postsurgical (+)	miniscrew (*n* = 71)	3 (elastic)	24	Pog 7.8			−0.9, 11.5%	NA
Korea													
Lai et al. [17]	IVRO	*n* = 41	21.5	28F	Presurgical (+)	No fixation	6 (wire)	13.3	Me 12.4	0.1, 0.8%	―		
2007			17–39	13M	Postsurgical (+)								
Taiwan													
Chung et al. [18]	SSRO	*n* = 60	22.3	34F	Presurgical (+)	miniplate (*n* = 30)	≤1 (elastic)	12	B point 8.76	−1.1, 12.6%	NA		
2008				26M	Postsurgical (+)				Pog 8.63	−2.26, 26.2%	NA		
Korea									Me 9.07	−1.94, 21.4%	NA		
						miniscrew (*n* = 30)	≤1 (elastic)	12	B point 6.97	−1.6, 23%	NA		
									Pog 8.03	−1.93, 24%	NA		
									Me 7.81	−2.26, 28.9%	NA		
Tseng et al. [19]	IVRO	*n* = 33	20.4	20F	Presurgical (+)	No fixation	6 (wire)	24	Me 12.4			−1, 8.1%	―
2016			17–34	13M	Postsurgical (+)								
Taiwan													
Schilbred Eriksen et al. [20]	IVRO	*n* = 36	21.6	24F	Presurgical (+)	No fixation	6 (wire)	150	B point 8.3			−1, 12%	*
2017			17.1–45.6	12M	Postsurgical (+)				Pog 9.3			−1.3, 14%	*
Norway													

*n*: number of samples; NA: Not available *: Significant, *p* < 0.05; ―: no significant.

## Data Availability

The data used to support the findings of this study are included within the article. The data used to support the findings of this study are available from the corresponding author upon request.
